# Genome-Wide Association Study of Genetic Variants Associated with Lower Extremity Amputation Risk in Peripheral Artery Disease

**DOI:** 10.3390/ijms27083405

**Published:** 2026-04-10

**Authors:** Rajashekar Korutla, Tanisha Garg, Michael P. Wilczek, Elsie G. Ross, Saeed Amal

**Affiliations:** 1 College of Engineering, The Roux Institute, Northeastern University, Portland, ME 04101, USA; korutla.r@northeastern.edu; 2 Department of Bioengineering, College of Engineering, Northeastern University, Boston, MA 02120, USA; 3 College of Science, The Roux Institute, Northeastern University, Portland, ME 04101, USA; 4 Department of Surgery, Division of Vascular Surgery, San Diego School of Medicine, University of California, La Jolla, San Diego, CA 92037, USA

**Keywords:** peripheral artery disease, amputation, genome-wide association study, genetic risk factors, single nucleotide polymorphisms, Firth correction

## Abstract

Peripheral artery disease (PAD) is a global health burden affecting over 200 million individuals and is frequently complicated by limb-threatening ischemia, leading to major amputations. Despite known clinical risk factors, the genetic basis underlying amputation risk in PAD remains poorly defined. In this study, we performed a multi-pronged genome-wide association study (GWAS) to identify genetic variants associated with lower extremity amputation in patients with PAD, using data from the *All of Us* Research Program. Two analytical strategies were employed: a targeted GWAS using ClinVar variants on the full cohort and a comprehensive genome-wide association study using Allele Count/Allele Frequency (ACAF) data on a balanced subset of the cohort. The ClinVar analysis of 118,871 variants in 7558 PAD patients (405 with amputation, 7153 without) identified 3 suggestive associations with a genomic inflation factor of 1.046. The ACAF analysis of 7,784,837 quality-controlled variants in 804 balanced samples (399 cases, 405 controls) yielded 35 suggestive associations (*p* < 1 × 10^−5^) with a genomic inflation factor of 1.017. No variants achieved suggestive significance in both analyses. These findings highlight candidate loci for further validation and may inform future development of risk prediction tools and targeted interventions to reduce limb loss in PAD. All associations are exploratory and require independent replication.

## 1. Introduction

Peripheral artery disease (PAD) represents a significant global health burden, affecting over 200 million individuals worldwide and increasing in prevalence with age [1,2]. PAD is characterized by atherosclerotic narrowing of the peripheral arteries, predominantly in the lower extremities, leading to reduced blood flow and tissue ischemia [3,4]. According to the 2017 ESC Guidelines, PAD is a progressive, systemic condition that requires comprehensive diagnostic and therapeutic strategies to reduce cardiovascular risk and prevent limb-threatening complications [5]. Similarly, the 2016 AHA/ACC guidelines emphasize early detection and comprehensive management of lower extremity PAD to reduce both cardiovascular and limb-related morbidity [6].

While many PAD patients remain asymptomatic or present with intermittent claudication, a subset progresses to critical limb ischemia (CLI), the most severe manifestation of PAD, carrying a high risk of limb loss [7]. Amputation represents a devastating complication of PAD that significantly impacts patient quality of life, functional status, and long-term survival [8]. Despite advances in revascularization techniques and medical therapy, amputation rates remain substantial, particularly among certain demographic groups and those with comorbidities such as diabetes mellitus [9]. Diabetes is a major risk factor for both the development and progression of PAD and significantly increases the likelihood of limb loss due to impaired vascular and metabolic responses [10,11]. The 5-year mortality rate following major amputation exceeds 50%, substantially higher than 5-year mortality observed in PAD patients without amputation, highlighting the severe prognostic implications of limb loss [7,12]. Moreover, patients with PAD often have polyvascular disease, which has been shown to significantly increase the risk of major cardiovascular events and may compound the risk of limb loss [13].

While clinical risk factors for PAD progression and amputation have been extensively studied, the genetic determinants underlying amputation risk remain poorly understood. Previous genetic studies in PAD have identified several loci associated with disease susceptibility and progression [14,15], but specific genetic variants predisposing to amputation have not been thoroughly investigated. Understanding the genetic architecture of amputation risk in PAD could inform future risk stratification approaches and generate hypotheses for therapeutic investigation. Despite advances in understanding PAD biology, the genetic underpinnings of disease susceptibility and progression remain incompletely defined, particularly for severe outcomes such as amputation [16].

Genome-wide association studies (GWASs) have emerged as a powerful approach for identifying genetic variants associated with complex traits and diseases [17]. This methodology has successfully uncovered numerous susceptibility loci for cardiovascular diseases, including coronary artery disease, stroke, and peripheral artery disease [18,19,20]. Previous GWASs in PAD have uniformly examined disease susceptibility comparing PAD patients to unaffected controls rather than disease progression to severe outcomes. Klarin et al. (2019) [20] performed a secondary analysis of Factor V Leiden against PAD severity subtypes in the Million Veteran Program, finding an increased risk of major amputation among carriers (OR = 1.62, *p* = 0.005), but this was limited to a single variant rather than a genome-wide investigation. Matsukura et al. (2015) [14] identified three susceptibility loci in a Japanese population without examining amputation as an outcome. Wassel et al. (2012) [15] and the CHARGE consortium [21] examined ankle-brachial index as a continuous trait. To our knowledge, no GWAS has used amputation as the primary phenotype in a genome-wide scan, nor employed a within-disease design comparing PAD patients with amputation to matched PAD controls without amputation. This distinction between susceptibility genetics (who develops PAD) and progression genetics (who progresses to amputation) is conceptually important, as the biological mechanisms driving disease complications may differ from those governing initial disease susceptibility.

Studies of disease progression within an affected population face unique statistical challenges distinct from traditional case–control susceptibility GWASs. Conditioning on disease status (PAD diagnosis) can introduce collider bias if shared genetic and environmental factors influence both disease susceptibility and severity [22,23]. Additionally, survival bias may arise if patients with the most aggressive genetic profiles do not survive to be enrolled, leading to underestimation of genetic effects. These considerations should be borne in mind when interpreting the results of progression GWAS, including the present study.

In this study, we conducted a comprehensive GWAS analysis to identify genetic variants associated with amputation in PAD patients. We employed multiple methodological approaches, including targeted analysis using the clinically relevant ClinVar dataset on the full cohort and a genome-wide analysis using the Allele Count/Allele Frequency (ACAF) dataset on a balanced subset of the cohort.

## 2. Results

### 2.1. ClinVar Analysis Results

The targeted GWAS using the ClinVar dataset did not identify any variants reaching genome-wide significance (*p* < 5 × 10^−8^). However, three variants showed suggestive associations (*p* < 1 × 10^−5^), as shown in Table 1.

The genomic inflation factor (λ) [24] was 1.046.

**Figure 1:**
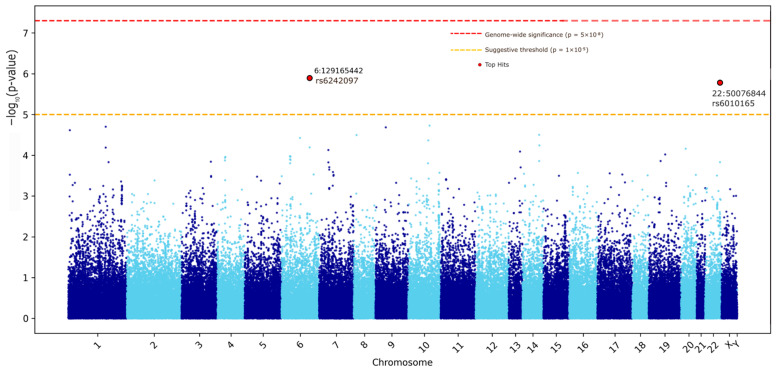
Manhattan Plot on Clinvar Analysis. Points are colored in alternating dark blue and sky blue to distinguish adjacent chromosomes.

**Figure 2:**
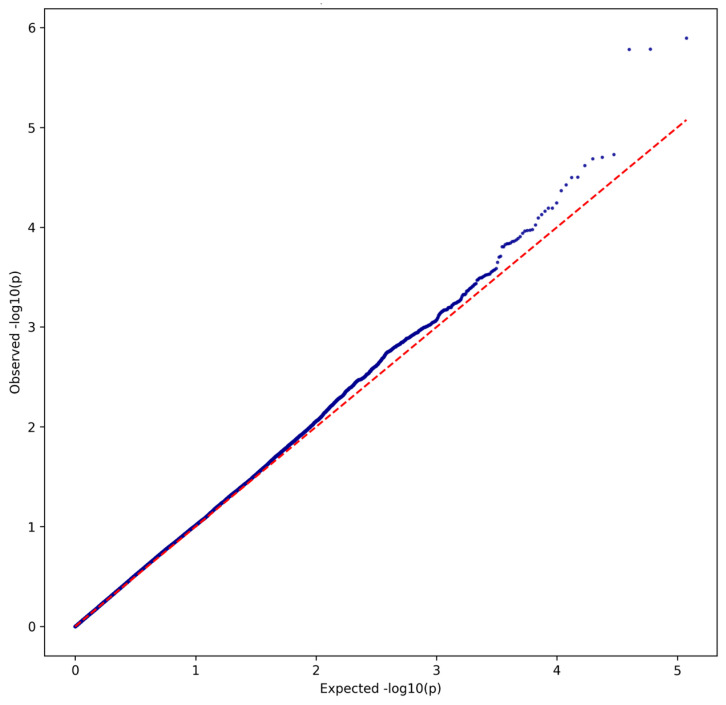
QQ Plot on Clinvar Analysis.

The Manhattan plot in Figure 1 shows peaks of suggestive significance, particularly on chromosomes 6 and 22, while the QQ plot in Figure 2 showed some deviation from the expected distribution at the tail end, suggesting potential associations.

### 2.2. ACAF Analysis Results

**Figure 3:**
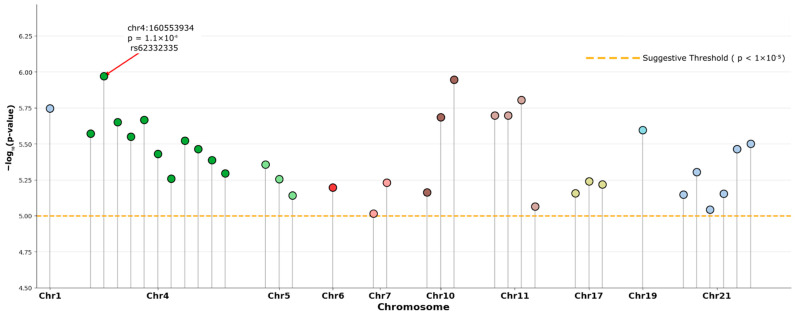
Manhattan Plot of ACAF analysis.

**Figure 4:**
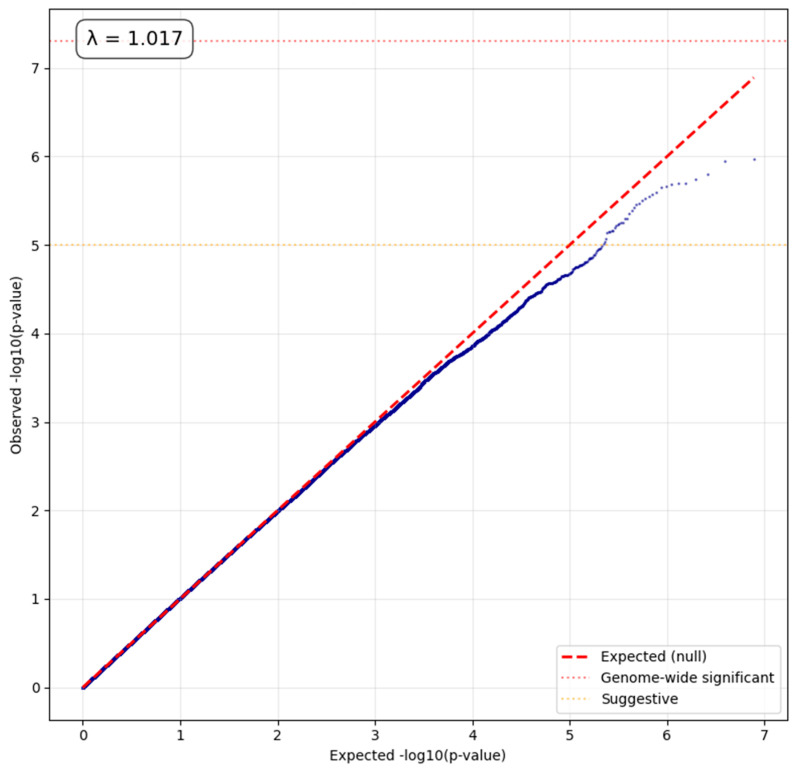
QQ Plot of ACAF analysis. The deviation at the upper right tail suggests there are some true genetic signals in the data.

The comprehensive GWAS using the ACAF dataset tested 7,784,837 variants in 804 samples. No variants reached genome-wide significance. 35 variants showed suggestive associations (*p* < 1 × 10^−5^). The genomic inflation factor (λ) was 1.017. The suggestive variants can be seen in Table 2, corresponding Manhattan and QQ plots are presented in Figure 3 and Figure 4.

### 2.3. Functional and Regulatory Annotation of Suggestive Variants

Comprehensive functional annotation of all 38 suggestive variants was performed using multiple bioinformatics platforms as mentioned in the Methods Section 4.6.

**Figure 5:**
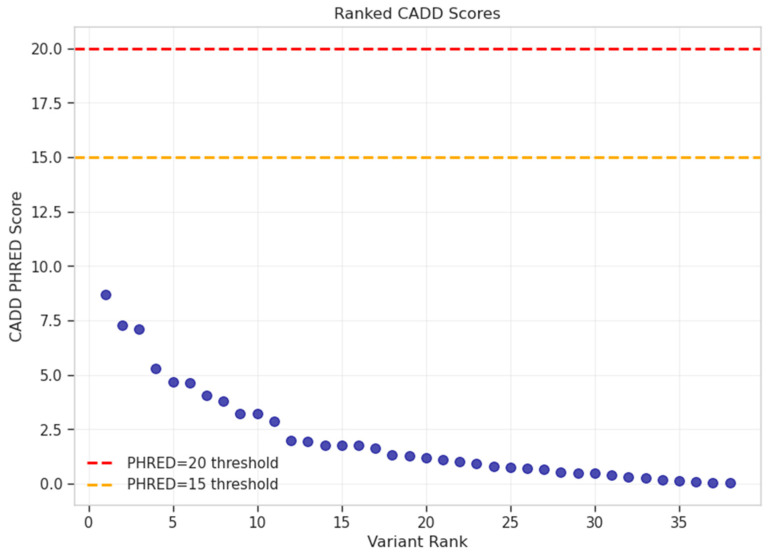
Ranked CADD PHRED scores for 38 PAD-associated variants, indicated by blue dots, showing all scores below pathogenicity thresholds (PHRED = 15 orange line, PHRED = 20 red line), with maximum score of 8.66.

**Figure 6:**
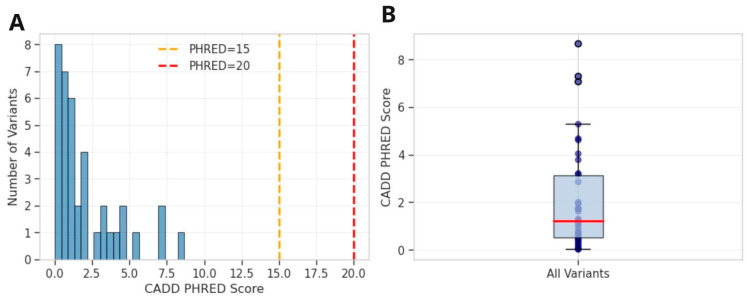
Distribution of CADD pathogenicity scores. (**A**): Histogram suggesting all variants cluster below clinical significance thresholds. (**B**): Box plot showing median PHRED score of 1.23 (IQR: 0.50–3.12) with all variants in the benign range.

The 38 variants displayed a characteristic complex-trait GWAS architecture dominated by non-coding variation. VEP analysis classified 94.7% of variants as non-coding: 44.7% intergenic (17/38), 34.2% intronic (13/38), with the remainder comprising upstream gene variants (3/38), 3′ UTR variants (2/38), and one each of 5′ UTR, synonymous, and splice region variant. CADD v1.7 PHRED scores were uniformly low (mean 2.06 ± 2.20, maximum 8.66), well below the threshold of 20 typically used to identify potentially pathogenic variants (Figure 5 and Figure 6A,B). The splice region variant rs267607236 in *MLC1* was evaluated using SpliceAI v1.3.1, which predicted no significant splicing impact (maximum delta score = 0.06, below the 0.2 threshold). Population frequency analysis via gnomAD v4 confirmed that 37/38 variants (97.4%) were common (MAF ≥ 5%), with a mean allele frequency of 30.96% and median of 29.60%. These 37 variants meet the ACMG BA1 criterion for benign classification in Mendelian contexts, though this does not preclude a role in complex trait susceptibility through regulatory mechanisms. The sole low-frequency variant was rs62420977 (chr6:129165442G>A, MAF = 2.15%), which showed notable enrichment in the Amish population (13.71%) compared to Non-Finnish European (2.95%), African (0.47%), and East Asian (0.02%) populations.

Expression quantitative trait loci analysis via GTEx v10 was limited by database coverage, with only 7/38 variants (18.4%) present. Three variants demonstrated significant QTL effects: rs6010165 (*MLC1*) showed a significant blood eQTL (*p* = 2.9 × 10^−13^, NES = −0.18), affecting expression of *MLC1*, *MOV10L1*, and *PANX2*, with additional splicing QTL activity; rs62420977 (*LAMA2*) showed a splicing QTL in thyroid tissue (*p* = 1.8 × 10^−5^, NES = −0.56); and rs2460694 (*B3GAT2*) showed a nominal eQTL in tibial nerve (*p* = 3.5 × 10^−4^, NES = 0.13). Critically, no variants showed eQTL effects in arterial tissues (aorta, coronary, tibial arteries). Regulatory element annotation was similarly limited: only one variant (rs58661519) overlapped with an annotated enhancer element; RegulomeDB analysis was successful for 15/38 variants (39.5%), with two (rs1045164134 and rs6010165) achieving score 1f indicating strong regulatory evidence; ENCODE SCREEN identified no candidate cis-regulatory elements at queried positions across 1518 biosamples; and available ENCODE chromatin accessibility data were restricted to five non-vascular cell types (HCT116 cells, activated and resting T-cells, A673 cells, and LPS-treated macrophages), preventing tissue-specific interpretation relevant to PAD. The complete absence of vascular-specific functional genomics data represents a critical gap in the interpretation of these findings.

### 2.4. Comparison of Results Across Analyses

#### 2.4.1. Genetic Architecture at Associated Loci

The integration of multiple analytical approaches suggested a consistent genetic architecture underlying PAD amputation risk. Conditional analysis across seven genomic regions containing multiple suggestive variants did not reveal evidence of secondary signals. These regions encompassed substantial genomic territory, with variant counts ranging from 682 in the chromosome 17 region to 1056 in the chromosome 11 region. After conditioning on the lead variant in each region, all association signals were effectively eliminated, with conditional *p*-values showing dramatic increases typically of several orders of magnitude compared to the original associations.

This pattern held true even for the chromosome 4 region at 167.3–167.4 Mb, which contained nine of our suggestive variants representing the highest density in our dataset. The complete elimination of secondary signals across all regions is consistent with each associated locus contains a single associated signal, with all other associations merely reflecting linkage disequilibrium. This relatively limited evidence of allelic heterogeneity (though power is limited) contrasts with many complex traits that show extensive allelic heterogeneity and suggests that identifying causal variants through fine mapping may be more tractable than initially anticipated.

#### 2.4.2. Impact of Study Design on Association Results

**Figure 7:**
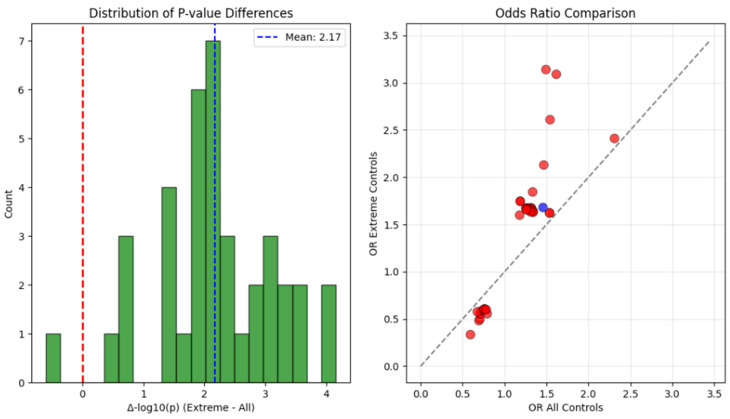
Alternative control analysis comparing extreme phenotype design versus all PAD controls. (**Left**): Distribution of *p*-value differences (Δ-log_10_(*p*)) showing 37/38 variants with improved significance using extreme phenotype design (positive values), with mean improvement of 2.17 orders of magnitude. (**Right**): Odds ratio comparison suggesting systematic attenuation of effect sizes when using all controls, with red points indicating variants that lost significance and the single blue point (rs60516505) showing improved association with all controls.

The alternative control analysis provided empirical support of our extreme phenotype design, illustrating the sensitivity of genetic discovery to study design choices. When we compared our matched design utilizing 399 cases and 405 controls against an analysis using all available PAD patients without amputation as controls, comprising 399 cases and 7139 controls, the results were notable. Despite the 18-fold increase in control sample size, which traditional power calculations would suggest should enhance discovery, we observed a substantial reduction in statistical significance for most variants. The systematic loss of signal when using all controls versus our extreme phenotype design is visualized in Figure 7, suggesting how the traditional approach dilutes amputation-specific genetic effects.

Of the 38 variants that achieved suggestive significance in our extreme phenotype design, 36 variants lost this significance threshold when tested with all controls, representing a 94.7% failure rate. The mean *p*-values increased by 2.17 orders of magnitude, approximately 148-fold, while mean odds ratios attenuated from 1.42 to 1.16, representing a 14.6% reduction in apparent effect size. Several variants showed particularly dramatic deterioration in statistical evidence. The variant rs59299975 on chromosome 11 saw its *p*-value deteriorate from 2.00 × 10^−6^ to 0.028, a 14,000-fold increase that would lead to its complete dismissal in a conventional analysis. Similarly, rs57745152 shifted from 7.00 × 10^−6^ to 0.026, and rs1834436109 moved from 6.87 × 10^−6^ to 0.012.

Intriguingly, only one variant, rs60516505 on chromosome 19, showed modest improvement with the all-controls design, with its *p*-value changing from 2.54 × 10^−6^ to 6.44 × 10^−7^. This unique behavior suggests this variant may capture genetic risk for general PAD severity rather than the specific progression to amputation, highlighting how different study designs can capture distinct aspects of disease biology.

We note that this comparison is specific to our phenotype and covariate structure, and should not be interpreted as general evidence that extreme phenotype designs are universally superior to traditional case–control approaches. The observed attenuation of signals with all controls may reflect genuine dilution of amputation-specific genetic effects by including mild PAD cases, but could also be influenced by differences in covariate distributions, residual population stratification, or model stability between the two designs. Additionally, unbalanced designs with substantially more controls can improve power for detecting common-variant effects in some settings [25].

#### 2.4.3. Robustness to Demographic Confounding

The stability of our genetic findings after demographic adjustment provides partial reassurance regarding their robustness to confounding, though this does not constitute validation of biological relevance. Our covariate adjustment analysis successfully tested all 38 candidate variants in 859 individuals with complete phenotype and genotype data, comprising 424 amputation cases and 435 controls. When comparing models incorporating only ancestry principal components against models that additionally included age at enrollment, the results suggested strong consistency.

**Figure 8:**
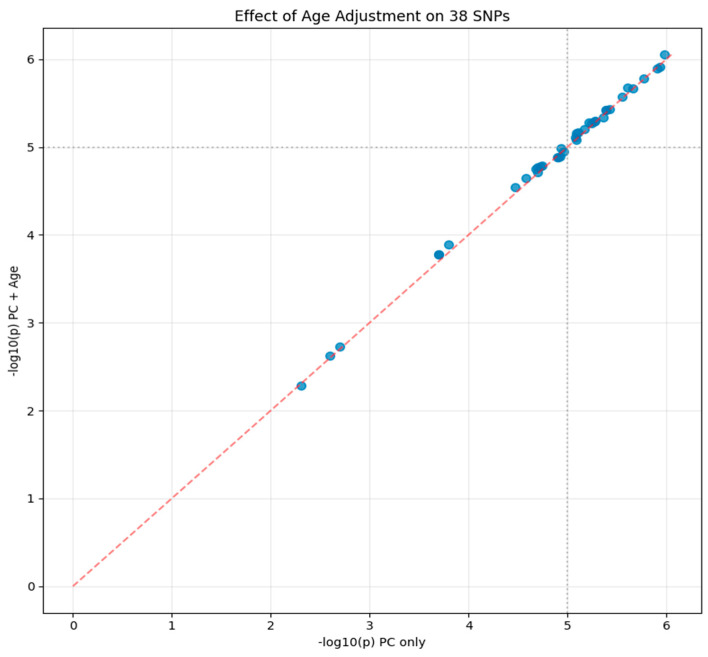
Impact of age adjustment on genetic associations for 38 suggestive variants. Each blue circle represents one variant, with −log_10_ (*p*-value) from the baseline model (ancestry principal components only) on the x-axis and −log_10_ (*p*-value) from the age-adjusted model (principal components plus age at enrollment) on the y-axis. The red dashed line represents the diagonal (y = x), indicating perfect concordance between models. Points clustering tightly along this line demonstrate that age adjustment had minimal impact on genetic associations (r = 0.999).

The *p*-values from the baseline and age-adjusted models showed near-perfect correlation with r = 0.999, indicating that age adjustment had minimal impact on the genetic associations. All 38 variants that showed suggestive association in the baseline model maintained this level of significance after age adjustment, with no variant losing its suggestive status. The mean -log_10_(*p*-value) across all variants increased slightly from 4.857 in the baseline model to 4.887 in the age-adjusted model, suggesting that accounting for age, if anything, slightly strengthened rather than weakened the genetic associations. The minimal impact of age adjustment on genetic associations is shown in Figure 8, with points clustering tightly along the diagonal.

Individual variants showed minimal change, with the strongest association at rs1256321231 in the *HTATIP2* gene improving from *p* = 1.04 × 10^−6^ to *p* = 8.89 × 10^−7^ after age adjustment. Among the top associations, the maximum change in *p*-value was less than one order of magnitude, with most variants showing changes of less than 10% in their −log_10_(*p*-value).

While we attempted to additionally adjust for sex, these models failed to converge due to numerical instability, likely reflecting the strong sex imbalance with approximately 62% male representation among amputation cases. However, given the extreme stability observed with age adjustment and the fact that ancestry principal components partially capture population-level sex differences, this limitation is unlikely to substantially impact our conclusions.

Sex-stratified genome-wide analyses were not performed because subdividing the sample of 804 individuals by sex would yield approximately 500 males and 300 females, resulting in critically underpowered subgroups for genome-wide testing. Based on our power calculations (Section 4.5), such analyses would only detect variants with OR ≥ 2.5 at MAF = 0.30 and genome-wide significance, substantially reducing sensitivity compared to the combined analysis.

### 2.5. Regional Architecture Patterns

**Figure 9:**
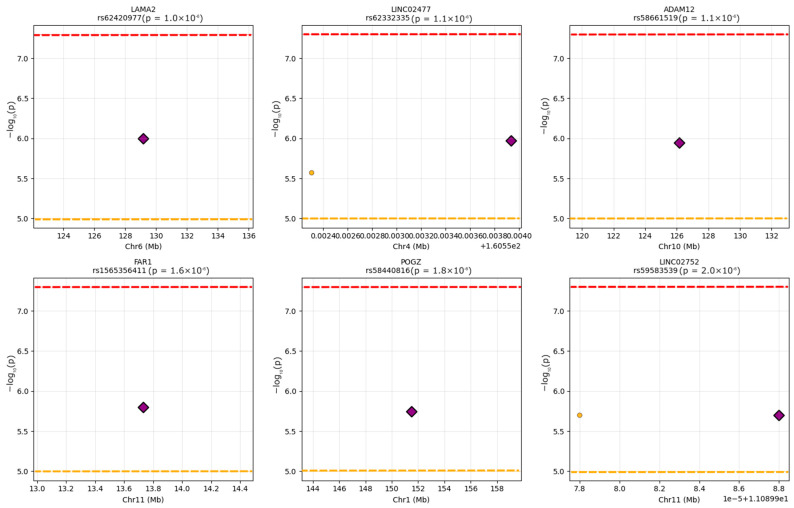
Regional association plots for the six most suggestive loci. Each panel displays a single locus with ±500 kb (or ±1 Mb for the chr4:167 Mb region) flanking the lead SNP. The lead variant is indicated by a purple diamond. Orange circles represent nearby variants within 10 kb of the lead SNP. The upper red dashed horizontal line denotes the genome-wide significance threshold (*p* = 5 × 10^−8^), and the lower orange dashed horizontal line denotes the suggestive significance threshold (*p* = 1 × 10^−5^).

The regional association analyses corroborated our conditional analysis findings while revealing interesting patterns of variant clustering. Analysis of the top six loci suggested that five regions showed isolated association peaks with minimal flanking signals, consistent with single causal variants driving the associations. The lead variants in these regions rose clearly above background noise with no accompanying signals approaching significance, supporting a limited evidence of allelic heterogeneity (though power is limited). Regional association plots for the six most suggestive loci are shown in Figure 9.

The *LINC02477* locus on chromosome 4 represented the sole exception to this pattern, displaying two variants that reached suggestive significance within approximately 1.6 kilobases of each other. These variants, rs62332335 at position 160,553,934 and rs377194319 at position 160,552,306, initially suggested possible allelic heterogeneity. Similarly, the *LINC02752* locus contained two variants (rs59299975 and rs59583539) separated by only 10 base pairs with identical association statistics, likely representing the same signal captured twice or variants in perfect linkage disequilibrium. Our conditional analysis suggested that both loci represent single association signals rather than independent effects.

The concentration of variants in certain genomic regions revealed intriguing patterns that warrant further investigation. Nine variants clustered near the pseudogene *RN7SL776P* on chromosome 4, with distances ranging from 26 to 44 kilobases, raising questions about whether this represents a regulatory hotspot affecting nearby genes or potentially reflects technical artifacts from repetitive sequences. Similarly, five variants associated with ENSG00000235965 on chromosome 21 suggest another potential regulatory domain. The identification of variants affecting long non-coding RNAs, specifically *LINC02477* and *LINC02752*, is consistent with emerging evidence for lncRNA involvement in vascular biology, though their specific functions remain largely uncharacterized.

## 3. Discussion

This genome-wide investigation of genetic determinants of amputation risk in PAD patients identified 38 suggestive loci using two complementary analytical strategies, none of which reached genome-wide significance. We emphasize that all 38 associations are suggestive (*p* < 1 × 10^−5^), non-replicated, and should be interpreted as hypothesis-generating. A substantial proportion of suggestive associations at this threshold are expected to be false positives given the number of tests performed.

### 3.1. Interpretation of Key Findings

The following gene-level interpretations should be considered hypothesis-generating, as the functional evidence for most variants is limited. All 38 suggestive variants had CADD PHRED scores below 10, 37 of 38 meet ACMG BA1 criteria for stand-alone benign classification based on allele frequency, and no variants showed eQTL effects in arterial tissues. Gene-level inferences are based primarily on genomic proximity and known biological roles rather than direct functional evidence linking these variants to PAD or amputation pathophysiology.

The absence of genome-wide significant findings with our limited sample size likely reflects limited statistical power rather than absence of genetic effects, given the observed effect sizes ranging from OR 0.48 to 3.14. The predominance of non-coding variants (94.7%) is consistent with the general pattern observed in GWAS of complex traits. Among our 38 variants, only five showed potential for direct functional impact: one splice region variant (rs267607236 in *MLC1*), one synonymous variant (rs6010165), two 3′ UTR variants (rs1565356411 in *FAR1* and rs57745152 in *NATD1*), and one 5′ UTR variant (rs1834436109 in *TUBB8*). However, computational predictions suggested limited functional consequences, with the *MLC1* splice variant showing SpliceAI scores below significance thresholds and all variants having CADD scores below 10.

The functional evidence was strongest for three variants confirmed as expression quantitative trait loci. The rs6010165 variant in *MLC1* showed a blood eQTL (*p* = 2.9 × 10^−13^) affecting multiple genes including *MLC1*, *MOV10L1*, and *PANX2*, though the relevance of this expression effect to PAD pathophysiology is unclear given the primary neural expression of *MLC1*. The rs62420977 variant in *LAMA2*, which encodes a vascular basement membrane component, showed splicing QTL activity in thyroid tissue (*p* = 1.8 × 10^−5^), though the functional significance for vascular biology remains speculative in the absence of arterial tissue data. Additionally, rs2460694 in *B3GAT2* showed a weak eQTL signal in tibial nerve (*p* = 3.5 × 10^−4^, NES = 0.13).

Several genes near our suggestive variants have biological roles that could speculatively relate to PAD progression, though no direct functional evidence links these specific variants to the implicated pathways. *ADAM12* encodes a metalloproteinase involved in extracellular matrix remodeling, *CUBN* is involved in vitamin B12 absorption potentially affecting homocysteine levels, and *FAR1* catalyzes fatty alcohol synthesis relevant to lipid metabolism. The identification of variants with protective direction of effect, such as rs58440816 near *POGZ* (OR = 0.56), raises the hypothesis that some genetic factors may confer resilience against progression to amputation, though this requires validation.

The high population frequencies observed 37 of 38 variants with MAF above 5% and a mean frequency of 31%, are inconsistent with Mendelian inheritance patterns. This frequency distribution, combined with uniformly low CADD scores, is consistent with a polygenic architecture where multiple common variants with individually small effects collectively influence amputation risk. However, because both analyses applied MAF filters (>0.05 for ACAF, >0.01 for ClinVar), this study was not designed to detect rare variant contributions, which may also play a role and would require gene-based burden or variance-component tests to evaluate.

### 3.2. Methodological Considerations

Our extreme phenotype design comparing amputation cases to matched PAD controls proved important for discovery. The alternative analysis suggested that using all 7139 available PAD patients as controls, despite providing 18-fold more samples, would have missed 94.7% of our associations. This likely occurs because including mild disease cases dilutes genetic signals specific to severe outcomes. Mean *p*-values deteriorated by 2.17 orders of magnitude in the conventional analysis, with some variants showing 14,000-fold increases in *p*-values.

The absence of secondary signals in conditional analysis across all seven tested regions simplifies interpretation. Each region appears to harbor a single association signal, with other associations reflecting linkage disequilibrium. The stability of associations after age adjustment (r = 0.999 between models) suggests these genetic factors operate independently of chronological age.

Post hoc power analysis confirmed that the ACAF cohort was adequately powered to detect large genetic effects (OR ≥ 1.8 at MAF ≥ 0.30) at genome-wide significance, but underpowered for the modest effect sizes observed for most suggestive variants (OR 1.60–1.68). Notably, three variants with the largest observed effect sizes rs17004490 (OR = 3.14), rs1565356411 (OR = 3.09), and rs1801996601 (OR = 2.61) would be expected to approach genome-wide significance based on power calculations, yet achieved only suggestive *p*-values. This discrepancy may reflect winner’s curse, whereby effect sizes in underpowered studies tend to be overestimated among variants crossing significance thresholds [26]. For comparison, the PAD GWAS by Klarin et al. (2019) [20] required over 31,000 cases to identify 19 genome-wide significant loci.

### 3.3. Biological and Clinical Implications

Our findings are consistent with a polygenic model where amputation risk may involve modest effects across multiple biological systems, though this interpretation is preliminary. The two eQTL findings *MLC1* in blood and *LAMA2* splicing in thyroid raise hypotheses involving systemic inflammation and vascular structural integrity, respectively, but neither has been validated in arterial tissue or in the context of PAD.

Clinical applications such as polygenic risk scores for amputation risk stratification are not justified by the current findings, which are based on non-replicated, sub-genome-wide associations that explain an unknown proportion of phenotypic variance. Should future large-scale studies confirm a subset of these loci, integration into polygenic risk frameworks could be explored, but this remains a distant goal requiring extensive validation. The population-specific enrichment of rs62420977 in Amish populations (13.7% vs. 2.15% in gnomAD exomes) illustrates the potential relevance of ancestry-specific allele frequency variation, though the clinical significance of this observation remains to be established.

### 3.4. Comparison with Previous Literature

As the first genome-wide scan using amputation as the primary phenotype within a PAD population, our findings have limited direct comparisons in the existing literature. The most relevant prior work is the PAD GWAS by Klarin et al. (2019) [20], which identified 19 susceptibility loci in over 31,000 MVP cases. Although their primary analysis compared PAD patients to unaffected controls without stratifying by severity, they performed a secondary analysis of Factor V Leiden across PAD severity subtypes, reporting an OR of 1.62 (*p* = 0.005) for major amputation. This single-variant finding represents the only prior genetic association with PAD-related amputation, but was not part of a genome-wide investigation. Matsukura et al. (2015) [14] identified susceptibility loci in a Japanese cohort without examining amputation. The Kullo et al. (2014) [27] eMERGE study examined PAD susceptibility using ABI-based phenotyping, also without amputation subanalysis.

The absence of overlap between our suggestive loci and previously reported PAD susceptibility variants is consistent with the hypothesis that progression genetics may differ from susceptibility genetics, as observed in other complex diseases. However, this lack of overlap could also reflect differences in statistical power, population composition, or phenotype definition between studies, and should not be overinterpreted.

The lack of genome-wide significant findings with our sample size mirrors early GWAS efforts in other vascular diseases [28,29]. Initial PAD GWAS studies similarly required meta-analyses of multiple cohorts to achieve genome-wide significance [21], suggesting our sample of 804 individuals represents an important first step requiring expansion through collaborative efforts.

### 3.5. Strengths and Limitations

This study’s primary strengths lie in its novel within-disease phenotype definition, specifically comparing PAD patients with amputation to matched PAD controls without amputation. The extreme phenotype design maximized statistical power from a moderate sample size. The *All of Us* cohort offers greater ancestral diversity than traditional European-focused studies [30]. Multiple sensitivity analyses provided a thorough evaluation of the robustness of findings.

The most critical limitation is the absence of independent replication. All 38 suggestive associations remain non-replicated, and at *p*-values on the order of 10^−6^ with millions of tests performed, a substantial proportion may represent false positives. Independent validation in an external cohort is essential before any biological or clinical conclusions can be drawn.

The amputation case definition did not distinguish between major and minor amputations, which may introduce phenotypic heterogeneity. Reliance on EHR-derived codes may result in misclassification. The requirement for whole-genome sequencing data restricted the ACAF analysis to 5.8% of the PAD cohort, and selection bias due to differential genomic data availability cannot be excluded. This study was not designed to detect rare variant associations (MAF < 0.01), which would require burden or variance-component tests [31]. Clinical covariates including diabetes, smoking, and hypertension were not incorporated into GWAS models due to convergence failures and concerns about collider bias when conditioning on potential mediators [22]; however, unmeasured confounding cannot be excluded. For example, identified variants could be associated with amputation indirectly through correlation with ancestry-specific diabetes susceptibility, smoking behavior, or differential access to vascular care, rather than through direct biological effects on limb ischemia. The clinical profile of this cohort has been characterized in detail elsewhere [11]. Functional annotation was constrained by the absence of vascular-specific data in public databases. The cross-sectional design prevented temporal analysis.

### 3.6. Future Directions

These findings establish a foundation for larger collaborative efforts needed to achieve genome-wide significance and enable robust replication. Functional validation of the two eQTL variants (rs6010165 and rs62420977) in disease-relevant cell types including vascular cells for *LAMA2* and blood/immune cells for *MLC1* represents a key priority. Development of vascular-specific functional genomics resources, including arterial eQTL datasets and endothelial cell chromatin accessibility maps, is critical for understanding these associations. Longitudinal studies tracking progression from PAD to amputation would clarify temporal relationships between genetic factors and clinical outcomes. Future studies with larger sample sizes should incorporate clinical covariates using methods robust to mediator adjustment, such as instrumental variable approaches or Mendelian randomization.

## 4. Materials and Methods

### 4.1. Study Population and Phenotype Definitions

The study cohort was drawn from the *All of Us* Research Program, a longitudinal cohort study with a goal of enrolling over one million participants across the United States and reflecting the diversity of the U.S. population [32]. Patient data were extracted from electronic health records (EHR) harmonized to the Observational Medical Outcomes Partnership (OMOP) Common Data Model (CDM) [33].

#### 4.1.1. PAD Case Identification

Patients with peripheral artery disease were identified using OMOP standard concept names queried from the condition_occurrence table. The concept set encompassed the full clinical spectrum of PAD, including atherosclerotic disease of the peripheral arteries (e.g., “Peripheral arterial disease,” “Atherosclerosis of artery of lower limb,” “Peripheral arterial occlusive disease”), symptomatic manifestations (e.g., “Intermittent claudication,” “Rest pain,” “Pain at rest due to peripheral vascular disease”), severe limb-threatening presentations (e.g., “Ischemic ulcer,” “Ulcer of lower extremity,” “Peripheral gangrene,” “Arteriosclerotic gangrene,” “Gangrene of limb due to atherosclerosis of artery of limb”), graft-related conditions (e.g., “Atherosclerosis of autologous vein bypass graft of limb,” “Disorder of vascular graft”), and diabetes-associated vascular disease (“Peripheral vascular disorder due to diabetes mellitus”). A total of 29 OMOP standard concept names were used to define the PAD cohort. The complete list of concept names is provided in Supplementary Table S1. This broad phenotyping strategy was adopted to capture the full clinical spectrum of PAD as represented in EHR coding practice, where the same underlying disease may be documented using different terminology depending on clinical stage, anatomical specificity, or care setting. This approach is consistent with standard methods for defining disease cohorts in observational research using the OMOP CDM [33] and identified 14,771 patients with at least one PAD-related condition record.

#### 4.1.2. Amputation Case Definition

Lower extremity amputation cases were identified using a curated set of OMOP standard concept IDs drawn from both the procedure_occurrence and condition_occurrence tables. The concept set was restricted to non-traumatic lower extremity amputations and included procedures at all anatomical levels (toe, foot, below-knee, above-knee, and hip disarticulation). Traumatic amputations were excluded by selecting only concept IDs corresponding to non-traumatic etiologies. A total of 103 OMOP concept IDs were used to identify amputation cases (see Supplementary Table S2 for the complete list). The concept IDs mapped to standard vocabularies including CPT4, ICD10PCS, SNOMED, and HCPCS.

#### 4.1.3. Temporal Criteria and Cohort Assembly

To ensure that amputations represented a consequence of PAD rather than an unrelated event, we required that each patient’s first recorded amputation date occurred after their first recorded PAD diagnosis date in the EHR. Patients were classified as amputation cases if they had at least one qualifying non-traumatic lower extremity amputation record following their initial PAD diagnosis. For patients with multiple amputation records (e.g., bilateral or staged procedures), only the first amputation event was used for case classification. All remaining PAD patients without a qualifying amputation record served as the non-amputation control pool.

This process identified 613 amputation cases (4.2%) and 14,158 non-amputation controls (95.8%) from the total PAD cohort of 14,771 patients.

### 4.2. Patient Cohorts

For the ClinVar analysis, we utilized the full cohort of 14,771 PAD patients identified in the *All of Us* database, comprising 613 patients who had undergone amputation and 14,158 who had not. Demographic data, including age and sex, were available for 9607 patients (65% of the cohort). After filtering for genetic data availability and complete covariate information, 7558 patients were included in the final analysis, comprising 405 amputation cases and 7153 controls.

For the ACAF analysis, we employed a balanced case–control design to optimize statistical power and computational efficiency. From the initial 14,771 PAD patients (613 with amputation [4.2%], 14,158 without amputation [95.8%]), we observed significant demographic differences between groups. Amputated patients were younger (median age 60 vs. 68 years), more likely to be male (63.4% vs. 50.2%), Black/African American (25.8% vs. 16.0%), or Hispanic/Latino (18.4% vs. 11.4%). To create a balanced cohort, we performed optimal one to one matching using the Hungarian algorithm [34] (linear sum assignment, implemented via SciPy v1.17.1, SciPy Community, https://scipy.org) based on Euclidean distances calculated from normalized features including age, race, ethnicity, sex, number of observations, and length of observation. Features were standardized using StandardScaler (scikit-learn v1.8.0, scikit-learn Community, https://scikit-learn.org) [35], and categorical variables were label-encoded. This ensured each non-amputation patient was matched to exactly one amputation patient, minimizing the overall matching distance. The matching quality was excellent with a mean distance of 0.29 (median: 0.21, max: 1.59). We verified that no patients appeared in both groups.

**Figure 10:**
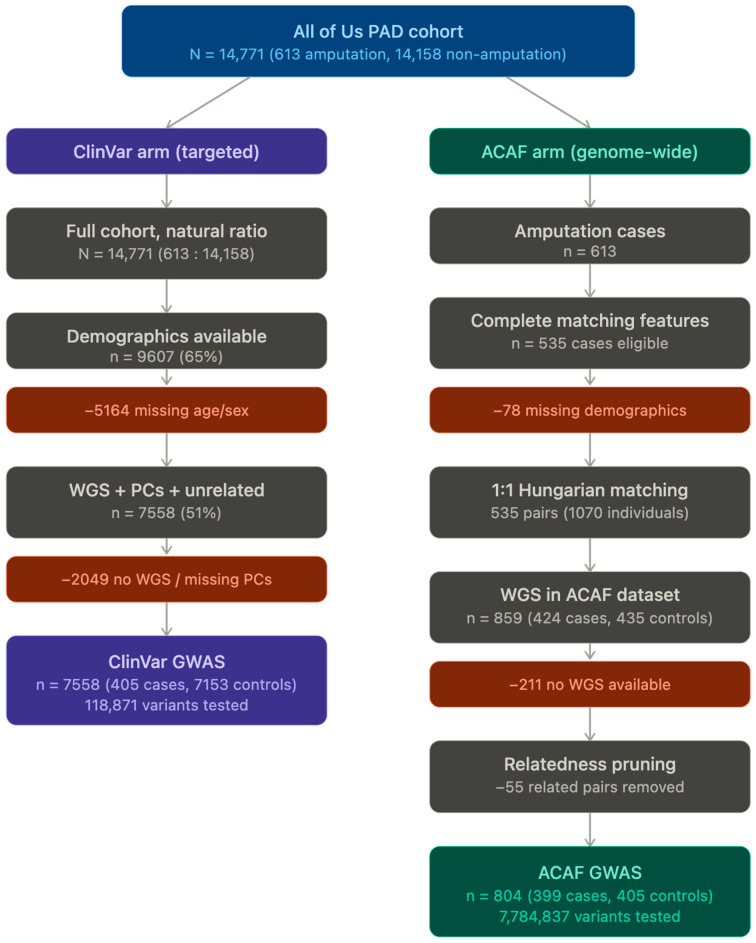
Cohort derivation flowchart for the ClinVar and ACAF analysis arms. Starting from 14,771 PAD patients identified in the *All of Us* Research Program, the diagram illustrates sample attrition through demographic filtering, whole-genome sequencing availability, Hungarian matching (ACAF arm only), and relatedness pruning. Final analyzed cohorts comprised 7558 individuals (405 cases, 7153 controls) for the ClinVar analysis and 804 individuals (399 cases, 405 controls) for the ACAF analysis.

From the 535 matched pairs, only 859 patients (5.8% of the original cohort) had whole genome sequencing data available in the ACAF dataset: 424 amputation cases and 435 controls. After removing related individuals, the final analysis cohort consisted of 804 individuals: 399 amputation cases and 405 controls. Cohort derivation for both analysis arms is summarized in Figure 10. The matching quality was assessed using standardized mean differences (SMD) across all matching variables as stated in Table 3. After 1:1 Hungarian matching, all SMDs were below 0.025, indicating excellent covariate balance between amputation cases and matched controls.

### 4.3. Genotyping and Quality Control

Genetic data were obtained from whole-genome sequencing performed as part of the All of Us Research Program.

#### 4.3.1. ClinVar Analysis

For the targeted analysis, we utilized the pre-processed ClinVar dataset, focusing on clinically relevant variants. Quality control procedures included filters for minor allele frequency (MAF > 0.01), Hardy–Weinberg equilibrium (*p* > 1 × 10^−10^), and call rate (>0.95). The initial ClinVar dataset contained 145,192 variants. Quality control removed 4662 variants with minor allele frequency ≤ 0.01 and 21,659 variants failing Hardy–Weinberg equilibrium (*p* ≤ 1 × 10^−10^), yielding 118,871 variants for analysis.

#### 4.3.2. ACAF Analysis

For the comprehensive genome-wide analysis, we employed the ACAF dataset version 8. Quality control procedures included:

- Call rate filter (>0.95): removed 31,671,940 variants.
- Minor allele frequency filter (>0.05): removed 76,514,080 variants.
- Hardy–Weinberg equilibrium filter (*p* > 1 × 10^−6^): removed 485,562 variants.
- Monomorphic variant check: removed 0 variants.

Starting with 116,456,419 variants, quality control procedures were applied sequentially. Yielding 7,784,837 variants for analysis. Related individuals were identified using the *All of Us* Research Program’s pre-computed relatedness flags (relatedness_flagged_samples.tsv), generated via the pc_relate method with a kinship coefficient threshold of approximately 0.1 (second-degree relatedness). From the 55 flagged related pairs, individuals were removed according to the default *All of Us* removal criteria without preferential retention of amputation cases, resulting in 804 (399 cases, 405 controls) individuals for analysis.

### 4.4. Statistical Analysis

All genome-wide association analyses were performed using Hail v0.2.130 [36] (Hail Team, Broad Institute of MIT and Harvard, Cambridge, MA, USA) running on Apache Spark v3.3.0 (Apache Software Foundation, Wilmington, DE, USA) within the All of Us Researcher Workbench (National Institutes of Health, Bethesda, MD, USA) running on Apache Spark 3.3.0 within the *All of Us* Researcher Workbench. We conducted two separate GWAS analyses with different designs based on data characteristics and computational constraints.

#### 4.4.1. ClinVar Analysis

Logistic regression was performed on 118,871 quality-controlled variants using Wald tests. Firth penalized logistic regression, which is recommended for rare variant analysis and severely imbalanced designs [37,38], was not employed for the primary ClinVar analysis because the MAF > 0.01 filter ensured adequate minor allele counts for standard logistic regression, and the analysis focused on a targeted set of clinically annotated variants rather than genome-wide testing. The initial cohort of 14,771 PAD patients was reduced to 7558 samples (51%) with complete data for all covariates after filtering for genetic data availability, demographic completeness, and relatedness. Covariates included age, sex, and the first three principal components of ancestry. The analysis utilized the natural case–control distribution (405 cases, 7153 controls) given the focused nature of testing pre-selected clinically relevant variants.

#### 4.4.2. ACAF Analysis

A comprehensive genome-wide analysis was performed on 7,784,837 quality-controlled variants using logistic regression with Wald tests. Initial attempts to include age and sex as covariates resulted in convergence failures, characterized by numerical overflow during Newton-Raphson iteration. This instability likely reflects quasi-complete separation, a well-documented phenomenon in logistic regression where a covariate (or combination of covariates) nearly perfectly predicts the outcome in a subset of observations, causing maximum likelihood estimates to diverge [39,40]. In the present study, the strong association between male sex and amputation status (63.4% male among cases vs. 50.2% among controls), combined with the modest sample size of 804 individuals and the need to fit models across 7,784,837 variants, created conditions where sparse covariate-outcome combinations in individual variant strata led to systematic convergence failure. After systematic evaluation, the final model included only the first three principal components of ancestry as covariates, achieving successful convergence across all variants. The balanced design (399:405) was specifically chosen to maximize statistical power and ensure computational stability when testing millions of variants.

We note that Firth penalized logistic regression, which addresses convergence issues due to separation by applying a bias-reduction penalty to the likelihood function [29], represents an alternative approach that could potentially accommodate additional covariates. However, the computational cost of applying Firth correction across 7,784,837 variants was prohibitive for this analysis. Our covariate adjustment analysis (Section “Covariate Adjustment Analysis”) demonstrated that the inclusion of age had minimal impact on genetic associations (r = 0.999 between models), providing partial reassurance that the ancestry-only model captures the primary confounding structure.

For both analyses:

- Statistical significance was defined as *p* < 5 × 10^−8^ for genome-wide significance and *p* < 1 × 10^−5^ for suggestive associations.
- Genomic inflation factor (λ) was calculated to assess potential population stratification or systematic biases.
- The results were visualized using Manhattan plots and quantile-quantile (QQ) plots.

### 4.5. Power Analysis

Post hoc power calculations were performed to characterize the detectable effect sizes under each study design, assuming an additive genetic model and 80% statistical power [41].

For the ACAF analysis (399 cases, 405 controls), the minimum detectable odds ratio at genome-wide significance (α = 5 × 10^−8^) varied with minor allele frequency: OR ≥ 3.5 at MAF = 0.05, OR ≥ 2.2 at MAF = 0.15, OR ≥ 1.8 at MAF = 0.30, and OR ≥ 1.7 at MAF = 0.50. At the suggestive threshold (α = 1 × 10^−5^), the corresponding minimum detectable odds ratios were OR ≥ 2.7, ≥ 1.8, ≥ 1.5, and ≥ 1.4, respectively. The majority of suggestive associations identified in this analysis had odds ratios between 1.60 and 1.68 at MAF values near 29%, placing them below the genome-wide detection threshold (OR ≥ 1.8) but within the suggestive detection range (OR ≥ 1.5), consistent with the observed pattern of suggestive but not genome-wide significant results.

The MAF filter of 0.05 in the ACAF analysis was motivated by statistical power: variants below this threshold would require odds ratios exceeding 3.5 for detection at genome-wide significance, an effect size uncommonly large for complex traits. Retaining such variants would increase the multiple testing burden without meaningful detection power.

For the ClinVar analysis (405 cases, 7153 controls), the extreme case–control imbalance (approximately 1:18) reduced the effective sample size to approximately 1533, calculated as N_eff = 4/(1/N_cases + 1/N_controls). Despite the 10-fold larger total sample, statistical power was only modestly greater than the balanced ACAF design. The more permissive MAF threshold of 0.01 was appropriate given both the larger effective sample and the substantially smaller number of variants tested (118,871 vs. 7,784,837), which reduced the multiple testing burden. At MAF = 0.02 (near the frequency of the top ClinVar hit rs62420977, MAF = 2.15%), the minimum detectable OR at genome-wide significance was approximately 3.2; the observed OR for this variant was 2.41, below this threshold, consistent with its suggestive but not genome-wide significant *p*-value (1.0 × 10^−6^).

Neither analysis was designed to detect associations with rare variants (MAF < 0.01), which would require gene-based burden or variance-component tests rather than single-variant association testing [31].

These calculations indicate that achieving 80% power for genome-wide detection of OR = 1.5 at MAF = 0.30 in a balanced design would require approximately 2800 cases, while detection of OR = 1.3 would require approximately 7500 cases, underscoring the need for collaborative multi-cohort efforts.

### 4.6. Bioinformatic Analysis

#### 4.6.1. Variant Annotation and Functional Prediction

All 38 suggestive variants (*p* < 1 × 10^−5^) from both ClinVar and ACAF analyses underwent comprehensive functional annotation using multiple bioinformatics resources.

##### Variant Effect Prediction

We annotated variants using the Ensembl Variant Effect Predictor (VEP) release 115 REST API (GRCh38/hg38; European Molecular Biology Laboratory–European Bioinformatics Institute (EMBL-EBI), Hinxton, Cambridge, UK) [42] to determine molecular consequences, affected genes, and gene biotypes. For intergenic variants, we identified the nearest gene within a 500 kb window using Ensembl’s overlap API. Complex variants such as deletions required alternative API query formats using dash notation for successful annotation retrieval.

##### Pathogenicity Assessment

We evaluated variant deleteriousness using the Combined Annotation Dependent Depletion (CADD) v1.7 (University of Washington, Seattle, WA, USA and Berlin Institute of Health at Charité, Berlin, Germany) [43] Both raw scores and PHRED-scaled scores were obtained, where PHRED scores of 10, 20, and 30 indicate variants in the top 10%, 1%, and 0.1% of deleterious variants, respectively.

##### Splicing Prediction

Splicing impact prediction was performed using SpliceAI v1.3.1 (Illumina, Inc., San Diego, CA, USA) [44] for variants annotated as splice region variants by VEP. SpliceAI delta scores were obtained for acceptor gain, acceptor loss, donor gain, and donor loss, with scores above 0.2 considered indicative of potential splicing effects.

##### Population Frequency Analysis

Allele frequencies across diverse populations were obtained from the Genome Aggregation Database (gnomAD v4) v4.1 (Broad Institute of MIT and Harvard, Cambridge, MA, USA) [45] via GraphQL API queries. Maximum allele frequencies were calculated across both exome and genome datasets for comprehensive coverage. Population-specific frequencies were assessed for European (EUR), African (AFR), East Asian (EAS), South Asian (SAS), and Latino/Admixed American (AMR) populations.

#### 4.6.2. Expression and Regulatory Analysis

##### Expression Quantitative Trait Loci (eQTL) Analysis

We queried the GTEx Portal v10 (Broad Institute of MIT and Harvard, Cambridge, MA, USA) [46] to identify whether variants act as expression or splicing quantitative trait loci. Each variant was searched by rsID to identify cis-eQTL effects (within 1 Mb of target genes) and splicing QTL effects across 54 tissues, with emphasis on vascular tissues (tibial artery, aorta), adipose tissue, and blood.

##### Regulatory Element Analysis

We employed two complementary approaches to identify regulatory variants. First, all variants were queried using RegulomeDB v2.2 (Stanford University, Stanford, CA, USA) [47], which integrates data from ENCODE, Roadmap Epigenomics, and other consortia, assigning categorical scores from 1a–7 based on regulatory evidence strength. Second, variants with the strongest RegulomeDB scores were validated using ENCODE SCREEN v3 (ENCODE Consortium, National Human Genome Research Institute, Bethesda, MD, USA) [48] to identify candidate cis-regulatory elements based on chromatin accessibility and histone modifications across 1518 biosamples.

##### Chromatin Accessibility Assessment

We queried the ENCODE database REST API (ENCODE Consortium, National Human Genome Research Institute, Bethesda, MD, USA) for DNase-seq and ATAC-seq experiments, prioritizing vascular-relevant cell types including endothelial cells, vascular smooth muscle cells, and monocytes. Regulatory features within ±5 kb of each variant were identified using the Ensembl Regulation database.

#### 4.6.3. Statistical Refinement Analyses

##### Conditional Analysis

To identify independent signals within associated loci, we performed stepwise conditional analysis on seven genomic regions containing multiple suggestive variants. Genotype data were extracted for expanded windows (100–200 kb) around each cluster, and association testing was repeated while adjusting for the lead variant genotype dosage and the same covariates used in the primary analysis.

##### Alternative Control Analysis

We evaluated the impact of control selection by comparing our extreme phenotype design (1:1 matched cases and controls) against a traditional approach using all available PAD patients without amputation as controls (1:17.9 ratio). Association statistics were compared between approaches for all 38 suggestive variants.

##### Covariate Adjustment Analysis

To assess robustness to demographic confounding, we compared models with increasing covariate complexity: Model 1 (baseline) with only ancestry principal components, and Model 2 with principal components plus age at enrollment. Additional models incorporating sex failed to converge due to numerical instability.

#### 4.6.4. Regional Visualization

For the six most significant loci, we generated regional association plots spanning 1–2 Mb windows centered on lead variants. Plots displayed −log_10_ (*p*-values) against chromosomal position, with variants colored by physical distance from the lead SNP as a proxy for linkage disequilibrium. Horizontal reference lines indicated genome-wide significance (*p* = 5 × 10^−8^) and suggestive significance (*p* = 1 × 10^−5^) thresholds. Gene annotations were extracted and filtered to display relevant protein-coding genes and long non-coding RNAs within each region.

## 5. Conclusions

This genome-wide association study represents an initial investigation of genetic factors specifically influencing amputation risk in peripheral artery disease patients. Through careful extreme phenotype design, we identified 38 suggestive genetic associations that may implicate diverse biological pathways in disease progression. While these variants did not achieve genome-wide significance and show limited functional annotation with current resources, the study design successfully isolated genetic factors specific to amputation risk rather than general PAD susceptibility. The absence of detectable secondary signals at associated loci, if confirmed, could facilitate future fine-mapping efforts, and the stability of associations after age adjustment suggests these genetic factors operate through mechanisms independent of chronological age. These results suggest that progression to amputation may involve genetic factors distinct from those governing initial disease susceptibility, supporting precision medicine approaches that consider disease stage. Larger collaborative studies, functional validation, and development of vascular-specific genomic resources represent critical next steps in translating these findings to improved clinical outcomes for PAD patients at risk of limb loss.

## Figures and Tables

**Table 1:** 3 Suggestive SNPs from Clinvar analysis.

Chr:Position	rsID	Ref > Alt	Beta	*p*-Value	OR	Gene/Nearest Gene	Consequence	Distance (bp)	MAF (%)	eQTL	CADD PHRED
chr6:129165442	rs62420977	G > A	0.88	1.00 × 10^−6^	2.41	*LAMA2*	intron_variant	0	2.15	Yes	0.4
chr22:50076844	rs6010165	G > A	0.484	2.00 × 10^−6^	1.62	*MLC1*	synonymous_variant	0	11.4	Yes	0.54
chr22:50076841	rs267607236	T > C	0.484	2.00 × 10^−6^	1.62	*MLC1*	splice_region_variant	0	10.8	No	0.02

**Table 2:** Thirty-five Suggestive SNPs from ACAF analysis.

Chr:Position	rsID	Ref > Alt	Beta	*p*-Value	OR	Gene/Nearest Gene	Consequence	Distance (bp)	MAF (%)	eQTL (Presence)	CADD PHRED
chr4:160553934	rs62332335	G > C	−0.729	1.07 × 10^−6^	0.48	*LINC02477*	intron_variant	0	29.8	No	7.09
chr10:126157589	rs58661519	C > T	0.756	1.13 × 10^−6^	2.13	*ADAM12*	intron_variant	0	15.5	No	5.3
chr11:13732125	rs1565356411	TAA > T	1.128	1.58 × 10^−6^	3.09	*FAR1*	3_prime_UTR_variant	0	6.4	No	0.27
chr1:151481028	rs58440816	G > C	−0.588	1.80 × 10^−6^	0.56	*POGZ*	intergenic_variant	21,534	46.2	No	1.3
chr11:11089988	rs59583539	T > C	0.557	2.00 × 10^−6^	1.75	*LINC02752*	intron_variant	0	12.4	No	7.29
chr11:11089978	rs59299975	T > C	0.557	2.00 × 10^−6^	1.75	*LINC02752*	intron_variant	0	12.4	No	0.02
chr10:16831081	rs57775857	C > T	0.612	2.06 × 10^−6^	1.84	*CUBN*	intron_variant	0	33.9	No	2
chr4:167358505	rs10390166	A > T	0.517	2.17 × 10^−6^	1.68	*RN7SL776P*	intergenic_variant	41,990	30.6	No	4.06
chr4:167355663	rs58299204	G > A	0.515	2.23 × 10^−6^	1.67	*RN7SL776P*	intergenic_variant	44,832	29	No	1.03
chr19:7375984	rs60516505	A > G	0.519	2.54 × 10^−6^	1.68	*ARHGEF18-AS1, ARHGEF18*	intron_variant	0	27.4	No	0.08
chr4:160552306	rs377194319	T > TCTAA	−0.691	2.69 × 10^−6^	0.5	*LINC02477*	intron_variant	0	29.8	No	1.74
chr4:167358166	rs12108386	C > A	0.51	2.82 × 10^−6^	1.67	*RN7SL776P*	intergenic_variant	42,329	32.5	No	0.68
chr4:167372657	rs17601234	T > A	0.5	3.02 × 10^−6^	1.65	*RN7SL776P*	intergenic_variant	27,838	29	No	1.11
chr21:44596298	rs17004490	T > C	1.144	3.15 × 10^−6^	3.14	*TSPEAR, KRTAP10-7, KRTAP10-6*	intron_variant	0	14.9	No	4.69
chr4:167373408	rs111484533	A > ACAGT	0.494	3.43 × 10^−6^	1.64	*RN7SL776P*	intergenic_variant	27,087	31.1	No	2.88
chr21:19075049	rs143785792	G > A	−0.587	3.45 × 10^−6^	0.56	*ENSG00000235965*	intergenic_variant	27,365	37.2	No	3.79
chr4:167362902	rs60353699	G > T	0.504	3.73 × 10^−6^	1.66	*RN7SL776P*	intergenic_variant	37,593	29	No	1.62
chr4:167373717	rs59233454	G > A	0.491	4.13 × 10^−6^	1.63	*RN7SL776P*	intergenic_variant	26,778	29.1	No	1.77
chr5:133657373	rs59870923	T > C	0.516	4.40 × 10^−6^	1.68	*FSTL4*	intergenic_variant	44,832	51.8	No	3.21
chr21:19051267	rs1985175965	G > T	−0.534	5.00 × 10^−6^	0.59	*ENSG00000235965*	upstream_gene_variant	3583	33.8	No	0.32
chr4:167374290	rs58934032	C > T	0.488	5.09 × 10^−6^	1.63	*RN7SL776P*	intergenic_variant	26,205	29	No	1.77
chr4:167367142	rs59244366	T > C	0.492	5.52 × 10^−6^	1.64	*RN7SL776P*	intergenic_variant	33,353	29	No	8.66
chr5:133661151	rs1182945568	T > C	0.515	5.56 × 10^−6^	1.67	*FSTL4*	intergenic_variant	48,610	52.8	No	0.75
chr17:21269327	rs2508018174	G > A	−0.504	5.76 × 10^−6^	0.6	*ENSG00000289453*	intergenic_variant	6570	26.6	No	0.46
chr7:73141070	rs1801996601	C > CCACA	0.959	5.92 × 10^−6^	2.61	*SPDYE10*	intron_variant	0	15.9	No	0.12
chr17:21269385	rs1045164134	A > AT	−0.502	6.01 × 10^−6^	0.61	*ENSG00000289453*	intergenic_variant	6512	24.4	No	1.17
chr6:70891807	rs2460694	T > C	−0.551	6.35 × 10^−6^	0.58	*B3GAT2*	intron_variant	0	30.1	Yes	0.18
chr10:49442	rs1834436109	AGCTCAGGTGTCCTT > A	−1.096	6.87 × 10^−6^	0.33	*TUBB8*	5_prime_UTR_variant	0	13.2	No	3.22
chr17:21240761	rs57745152	G > A	0.469	7.00 × 10^−6^	1.6	*NATD1, TMEM11-DT*	3_prime_UTR_variant	0	10.7	No	0.66
chr21:19056133	rs28806580	A > G	−0.509	7.05 × 10^−6^	0.6	*ENSG00000235965*	intergenic_variant	8449	26.4	No	0.78
chr21:19051108	rs9653673	G > A	−0.515	7.26 × 10^−6^	0.6	*ENSG00000235965*	upstream_gene_variant	3424	26.3	No	4.62
chr5:133664291	rs56555387	G > A	0.508	7.28 × 10^−6^	1.66	*FSTL4*	intergenic_variant	51,750	53.5	No	1.95
chr11:20368197	rs1256321231	T > A	0.505	8.68 × 10^−6^	1.66	*HTATIP2*	intron_variant	0	12.8	No	0.49
chr21:19051807	rs1568913903	T > TTA	−0.503	9.12 × 10^−6^	0.6	*ENSG00000235965*	upstream_gene_variant	4123	26.4	No	1.28
chr7:37714274	rs1215147035	T > C	−0.513	9.65 × 10^−6^	0.6	*GPR141*	intron_variant	0	29.9	No	0.9

**Table 3:** Post-matching covariate balance between amputation cases and matched non-amputation controls in the ACAF analysis, assessed by standardized mean differences (SMD). All SMDs were below 0.025, indicating excellent balance across all matching variables.

Variable	Amputation Cases	Matched Controls	SMD
Age, mean ± SD	59.9 ± 11.5	60.1 ± 11.3	0.021
Male, %	63.1	63.1	0
Female, %	34.4	34.4	0
White, %	47.3	47.6	0.007
Black or African American, %	25.6	25.4	0.004
Asian, %	2	2	0
Hispanic or Latino, %	19.9	19.9	0
Not Hispanic or Latino, %	74.6	74.7	0.004
N observations, median	545	532	0.025

## Data Availability

The original contributions presented in this study are included in this article. Further inquiries can be directed to the corresponding author. The linked genotype and phenotype data analyzed in this study are from the National Institutes of Health’s *All of Us* Research Program and are available through its controlled-access Researcher Workbench. Due to the terms of the Data Use Agreement designed to protect participant privacy and data security, the raw and individual-level data cannot be downloaded or deposited into external public repositories. The full summary-level statistics from our genome-wide association study are available upon reasonable request to the corresponding author. All qualified researchers can apply for access to the same raw dataset by registering and completing the required ethics training via the *All of Us* Research Hub at https://www.researchallofus.org/.

## Supplementary Materials

The following supporting information can be downloaded at: https://www.mdpi.com/article/10.3390/ijms27083405/s1
