# Supplementary material for: Genome-Wide Association Study of Genetic Variants Associated with Lower Extremity Amputation Risk in Peripheral Artery Disease
Source: Int J Mol Sci. 2026 Apr 10;27(8):3405. doi: 10.3390/ijms27083405 (PMC13115880; doi:10.3390/ijms27083405)
Supplement: Supplementary file 1 [file ijms-27-03405-s001.zip › ijms-4170146-supplementary.pdf]

Supplementary Material:

Table S1:

Clinical Category	OMOP Standard Concept Name
**Atherosclerotic Disease of the Peripheral Arteries**	Peripheral arterial disease
	Peripheral arterial occlusive disease
	Atherosclerosis of artery of lower limb
	Atherosclerosis of arteries of the extremities
	Atherosclerosis of artery
	Atherosclerosis of aorta
	Arteriosclerotic vascular disease
	Chronic occlusion of artery of extremity
	peripheral artery
**Symptomatic Manifestations**	Intermittent claudication
	Intermittent claudication due to atherosclerosis of artery of limb
	Intermittent claudication of left lower limb co-occurrent and due to atherosclerosis
	Intermittent claudication of right lower limb co-occurrent and due to atherosclerosis
	Rest pain
	Pain at rest due to peripheral vascular disease
	Pain at rest of left lower limb co-occurrent and due to atherosclerosis
	Pain at rest of right lower limb co-occurrent and due to atherosclerosis
**Severe Limb-Threatening Presentations**	Ischemic ulcer
	Ulcer of lower extremity
	Ulcer of calf due to atherosclerosis of artery of lower limb

	Necrosis of bone of lower leg co-occurrent and due to chronic ulcer of lower leg
	Peripheral gangrene
	Arteriosclerotic gangrene
	Gangrene of limb due to atherosclerosis of artery of limb
**Graft-Related Conditions**	Disorder of vascular graft
	Atherosclerosis of bypass graft of limb
	Atherosclerosis of autologous vein bypass graft of limb
	Atherosclerosis of nonautologous biological bypass graft of limb
**Diabetes-Associated Vascular Disease**	Peripheral vascular disorder due to diabetes mellitus

Table S2:

Concept_ID	Concept_Name	Domain	Vocabulary
2105451	Amputation, ankle, through malleoli of tibia and fibula (eg, Syme, Pirogoff type procedures), with plastic closure and resection of nerves	Procedure	CPT4
2105804	Amputation, foot; midtarsal (eg, Chopart type procedure)	Procedure	CPT4
2105805	Amputation, foot; transmetatarsal	Procedure	CPT4
2105806	Amputation, metatarsal, with toe, single	Procedure	CPT4

2105209	Amputation, thigh, through femur, any level	Procedure	CPT4
2105210	Amputation, thigh, through femur, any level; immediate fitting technique including first cast	Procedure	CPT4
2105211	Amputation, thigh, through femur, any level; open, circular (guillotine)	Procedure	CPT4
2105223	Amputation, thigh, through femur, any level; re-amputation	Procedure	CPT4
2105222	Amputation, thigh, through femur, any level; secondary closure or scar revision	Procedure	CPT4
2105462	Ankle disarticulation	Procedure	CPT4
2105224	Disarticulation at knee	Procedure	CPT4
2784238	Anatomical Regions, Lower Extremities, Detachment	Procedure	ICD10PCS
2784478	Detachment at Left 1st Toe, Complete, Open Approach	Procedure	ICD10PCS
2784479	Detachment at Left 1st Toe, High, Open Approach	Procedure	ICD10PCS

2784481	Detachment at Left 1st Toe, Low, Open Approach	Procedure	ICD10PCS
2784480	Detachment at Left 1st Toe, Mid, Open Approach	Procedure	ICD10PCS
2784486	Detachment at Left 2nd Toe, Complete, Open Approach	Procedure	ICD10PCS
2784487	Detachment at Left 2nd Toe, High, Open Approach	Procedure	ICD10PCS
2784489	Detachment at Left 2nd Toe, Low, Open Approach	Procedure	ICD10PCS
2784488	Detachment at Left 2nd Toe, Mid, Open Approach	Procedure	ICD10PCS
2784494	Detachment at Left 3rd Toe, Complete, Open Approach	Procedure	ICD10PCS
2784495	Detachment at Left 3rd Toe, High, Open Approach	Procedure	ICD10PCS
2784497	Detachment at Left 3rd Toe, Low, Open Approach	Procedure	ICD10PCS
2784496	Detachment at Left 3rd Toe, Mid, Open Approach	Procedure	ICD10PCS

2784502	Detachment at Left 4th Toe, Complete, Open Approach	Procedure	ICD10PCS
2784503	Detachment at Left 4th Toe, High, Open Approach	Procedure	ICD10PCS
2784505	Detachment at Left 4th Toe, Low, Open Approach	Procedure	ICD10PCS
2784504	Detachment at Left 4th Toe, Mid, Open Approach	Procedure	ICD10PCS
2784510	Detachment at Left 5th Toe, Complete, Open Approach	Procedure	ICD10PCS
2784511	Detachment at Left 5th Toe, High, Open Approach	Procedure	ICD10PCS
2784513	Detachment at Left 5th Toe, Low, Open Approach	Procedure	ICD10PCS
2784512	Detachment at Left 5th Toe, Mid, Open Approach	Procedure	ICD10PCS
2784243	Detachment at Left Femoral Region, Open Approach	Procedure	ICD10PCS
2784251	Detachment at Left Knee Region, Open Approach	Procedure	ICD10PCS

2784247	Detachment at Left Upper Leg, High, Open Approach	Procedure	ICD10PCS
2784249	Detachment at Left Upper Leg, Low, Open Approach	Procedure	ICD10PCS
2784248	Detachment at Left Upper Leg, Mid, Open Approach	Procedure	ICD10PCS
2784474	Detachment at Right 1st Toe, Complete, Open Approach	Procedure	ICD10PCS
2784475	Detachment at Right 1st Toe, High, Open Approach	Procedure	ICD10PCS
2784477	Detachment at Right 1st Toe, Low, Open Approach	Procedure	ICD10PCS
2784476	Detachment at Right 1st Toe, Mid, Open Approach	Procedure	ICD10PCS
2784482	Detachment at Right 2nd Toe, Complete, Open Approach	Procedure	ICD10PCS
2784483	Detachment at Right 2nd Toe, High, Open Approach	Procedure	ICD10PCS
2784485	Detachment at Right 2nd Toe, Low, Open Approach	Procedure	ICD10PCS

2784484	Detachment at Right 2nd Toe, Mid, Open Approach	Procedure	ICD10PCS
2784490	Detachment at Right 3rd Toe, Complete, Open Approach	Procedure	ICD10PCS
2784491	Detachment at Right 3rd Toe, High, Open Approach	Procedure	ICD10PCS
2784493	Detachment at Right 3rd Toe, Low, Open Approach	Procedure	ICD10PCS
2784492	Detachment at Right 3rd Toe, Mid, Open Approach	Procedure	ICD10PCS
2784498	Detachment at Right 4th Toe, Complete, Open Approach	Procedure	ICD10PCS
2784499	Detachment at Right 4th Toe, High, Open Approach	Procedure	ICD10PCS
2784501	Detachment at Right 4th Toe, Low, Open Approach	Procedure	ICD10PCS
2784500	Detachment at Right 4th Toe, Mid, Open Approach	Procedure	ICD10PCS
2784506	Detachment at Right 5th Toe, Complete, Open Approach	Procedure	ICD10PCS

2784507	Detachment at Right 5th Toe, High, Open Approach	Procedure	ICD10PCS
2784509	Detachment at Right 5th Toe, Low, Open Approach	Procedure	ICD10PCS
2784508	Detachment at Right 5th Toe, Mid, Open Approach	Procedure	ICD10PCS
2784242	Detachment at Right Femoral Region, Open Approach	Procedure	ICD10PCS
2784250	Detachment at Right Knee Region, Open Approach	Procedure	ICD10PCS
2784244	Detachment at Right Upper Leg, High, Open Approach	Procedure	ICD10PCS
2784246	Detachment at Right Upper Leg, Low, Open Approach	Procedure	ICD10PCS
2784245	Detachment at Right Upper Leg, Mid, Open Approach	Procedure	ICD10PCS
2858313	Medical and Surgical @ Anatomical Regions, Lower Extremities @ Detachment @ Femoral Region, Left	Procedure	ICD10PCS

2866618	Medical and Surgical @ Anatomical Regions, Lower Extremities @ Detachment @ Femoral Region, Left @ Open	Procedure	ICD10PCS
2898474	Medical and Surgical @ Anatomical Regions, Lower Extremities @ Detachment @ Femoral Region, Right	Procedure	ICD10PCS
2858312	Medical and Surgical @ Anatomical Regions, Lower Extremities @ Detachment @ Femoral Region, Right @ Open	Procedure	ICD10PCS
2845039	Medical and Surgical @ Anatomical Regions, Lower Extremities @ Detachment @ Hindquarter, Bilateral	Procedure	ICD10PCS
2815480	Medical and Surgical @ Anatomical Regions, Lower Extremities @ Detachment @ Hindquarter, Bilateral @ Open	Procedure	ICD10PCS
2815479	Medical and Surgical @ Anatomical Regions, Lower Extremities @ Detachment @ Hindquarter, Left	Procedure	ICD10PCS
2852770	Medical and Surgical @ Anatomical Regions, Lower Extremities @ Detachment @ Hindquarter, Left @ Open	Procedure	ICD10PCS
2871784	Medical and Surgical @ Anatomical Regions, Lower Extremities @ Detachment @ Hindquarter, Right	Procedure	ICD10PCS

2866617	Medical and Surgical @ Anatomical Regions, Lower Extremities @ Detachment @ Hindquarter, Right @ Open	Procedure	ICD10PCS
2006242	Amputation of ankle through malleoli of tibia and fibula	Procedure	ICD9Proc
3173821	Amputation of all right toes	Procedure	Nebraska Lexicon
3168091	Amputation of toes 1 through 4 left foot	Procedure	Nebraska Lexicon
4195136	Amputation above-knee	Procedure	SNOMED
4002166	Amputation above-knee, mid-thigh	Procedure	SNOMED
4054498	Amputation below-knee conversion into above-knee amputation	Procedure	SNOMED
4217266	Amputation by disarticulation of hindfoot	Procedure	SNOMED
4108567	Amputation lesser toe	Procedure	SNOMED
4264289	Amputation of ankle	Procedure	SNOMED
4218050	Amputation of hallux	Procedure	SNOMED
37115743	Amputation of left lower limb	Procedure	SNOMED

36717437	Amputation of left lower limb above knee	Procedure	SNOMED
4338257	Amputation of leg through tibia and fibula	Procedure	SNOMED
4266202	Amputation of limb	Procedure	SNOMED
4219032	Amputation of lower limb	Procedure	SNOMED
4078563	Amputation of phalanx of toe	Procedure	SNOMED
37118455	Amputation of right lower limb	Procedure	SNOMED
36715395	Amputation of right lower limb above knee	Procedure	SNOMED
4108565	Amputation of the foot	Procedure	SNOMED
4159766	Amputation of toe	Procedure	SNOMED
4302020	Amputation of toe at interphalangeal joint	Procedure	SNOMED
4272232	Amputation of toe at metatarsophalangeal joint	Procedure	SNOMED
4054983	Amputation through foot	Procedure	SNOMED
4106053	Disarticulation - action	Observation	SNOMED
4177620	Lower thigh amputation	Procedure	SNOMED

37116276	Partial amputation of right toe	Procedure	SNOMED
37204044	Partial amputation of toe of left foot	Procedure	SNOMED
4119910	Supramalleolar ankle amputation	Procedure	SNOMED
4169822	Syme through ankle amputation	Procedure	SNOMED
4143795	Through knee amputation	Procedure	SNOMED
4183102	Upper thigh amputation	Procedure	SNOMED
